# Lipid Bilayer Membrane in a Silicon Based Micron Sized Cavity Accessed by Atomic Force Microscopy and Electrochemical Impedance Spectroscopy

**DOI:** 10.3390/bios7030026

**Published:** 2017-07-05

**Authors:** Muhammad Shuja Khan, Noura Sayed Dosoky, Darayas Patel, Jeffrey Weimer, John Dalton Williams

**Affiliations:** 1 Electrical and Computer Engineering Department, University of Alabama in Huntsville, Huntsville, AL 35899, USA; john@johndwilliams.org; 2 Biotechnology Science and Engineering Program, University of Alabama in Huntsville, Huntsville, AL 35899, USA; noura.dosoky@vanderbilt.edu; 3 Department of Mathematics and Computer Science, Oakwood University, Huntsville, AL 35896, USA; dpatel@oakwood.edu; 4 Chemistry/Chemical and Materials Engineering Department, University of Alabama in Huntsville, Huntsville, AL 35899, USA; weimerj@uah.edu

**Keywords:** lipid bilayer membrane, large unilamellar vesicles, silicon cavity, atomic force microscopy, electrochemical impedance spectroscopy.

## Abstract

Supported lipid bilayers (SLBs) are widely used in biophysical research to probe the functionality of biological membranes and to provide diagnoses in high throughput drug screening. Formation of SLBs at below phase transition temperature (*Tm*) has applications in nano-medicine research where low temperature profiles are required. Herein, we report the successful production of SLBs at above—as well as below—the *Tm* of the lipids in an anisotropically etched, silicon-based micro-cavity. The Si-based cavity walls exhibit controlled temperature which assist in the quick and stable formation of lipid bilayer membranes. Fusion of large unilamellar vesicles was monitored in real time in an aqueous environment inside the Si cavity using atomic force microscopy (AFM), and the lateral organization of the lipid molecules was characterized until the formation of the SLBs. The stability of SLBs produced was also characterized by recording the electrical resistance and the capacitance using electrochemical impedance spectroscopy (EIS). Analysis was done in the frequency regime of 10^−2^–10^5^ Hz at a signal voltage of 100 mV and giga-ohm sealed impedance was obtained continuously over four days. Finally, the cantilever tip in AFM was utilized to estimate the bilayer thickness and to calculate the rupture force at the interface of the tip and the SLB. We anticipate that a silicon-based, micron-sized cavity has the potential to produce highly-stable SLBs below their *Tm*. The membranes inside the Si cavity could last for several days and allow robust characterization using AFM or EIS. This could be an excellent platform for nanomedicine experiments that require low operating temperatures.

## 1. Introduction

Supported lipid bilayers (SLBs) are commonly used to model cell membranes and its native proteins [1]. Production of SLBs as a model system comprised of either single or multiple component lipids has been done using vesicle fusion [2,3,4,5,6,7], Langmuir–Blodgett/Langmuir–Schaefer deposition [8,9]. Scanning probe lithography methods as Dip-Pen Nanolithography [10,11,12,13] and Polymer Pen Lithography [14] could also be utilized for the production of lipid bilayer membrane. Vesicle fusion is the most common method on hydrophilic surfaces [1]. Vesicles adsorb on the surface and rupture, leading to bilayer formation across the entirety of the hydrophilic support [15]. The presence of a hydration layer approximately 1–2 nm between the support and the lower lipid leaflet allows the bilayer to remain laterally fluid and enables diffusion of lipids [16]. The production of SLBs is often carried out at above the phase transition temperature (*Tm*) of lipids on supports such as glass, mica, Si3N4, and porous membranes including alumina, gold coated alumina, and silicon (Si) [1]. In recent years, the scientific community has shown a progressive interest in analyzing biological samples on silicon based materials because they have proven to be biocompatible, biodegradable, and photoluminescent [1,17,18]. Few reports are available for the production of lipid membranes below or close to their *Tm* [19,20]. The protocols used to prepare membranes on substrates also vary [21,22,23,24], possibly due to variations in the concentration of the lipids, in their incubation temperature and time, or in the substrates used. The transition temperature is expected to depend on the vesicle size or the substrate. However, the formation of lipid bilayers at or in proximity to *Tm* has been rarely studied to validate this hypothesis. One study has been reported with 1,2-dipalmitoyl-sn-glycero-3-phosphocholine (DPPC) mixtures [19], and the parameters governing the process have only been partially discussed.

Atomic force microscopy (AFM) has been widely utilized to investigate the mechanical behavior of SLBs at real space under physiological environment [25]. With AFM, vesicles and bilayer patches can easily be distinguished from each other at nanometer resolutions. Electrochemical impedance spectroscopy (EIS) has also been utilized to investigate the electrical properties (resistance and capacitance) of the pre-formed lipid bilayer membranes produced at different support [26]. Therefore, AFM and EIS are the potential tools to characterize the membrane formation process in real-time fashion at above and below the *Tm* of the lipids and also to investigate the stability of the membrane for longer duration. 

In this work, we present the complete, robust method to prepare SLBs at the center of an anisotropically etched, thin Si membrane. An etched well was created to maintain a controlled temperature for the buffer solution where the lipid bilayer can easily be formed. The procedure started with the layout that will produce a silicon micro-cavity using microfabrication. This cavity was used to create SLBs at temperatures above and below *Tm* of the lipids. The effect of incubation temperature and cooling rate on the quality of membrane formed by 1,2-dipalmitoyl-sn-glycero-3-phosphoethanolamine (DPPE) and 1,2-dipalmitoyl-sn-glycero-3-phosphoserine (DPPS) were elaborated and captured. Experiments have been done in a natural fluidic environment using AFM to monitor the continuous fusion of vesicles until the complete formation of the SLBs inside the cavity. The electrical resistance and the capacitance of the bilayers were obtained using EIS continuously for over four days. We finally demonstrate that rupture force and lipid bilayer depth values for SLB produced below the *Tm* by employing force spectroscopy experiment and the results are in good agreement with other AFM studies. 

## 2. Materials and Methods

### 2.1. Materials and Instruments

A silicon wafer polished on both side was purchased from Silicon Valley Microelectronics, Inc. (Santa Clara, CA, USA), stock ampules (25 mg) of 1,2-dipalmitoyl-sn-glycero-3-phosphoethanolamine (DPPE) and 1,2-dipalmitoyl-sn-glycero-3-phosphoserine (DPPS) were purchased from Avanti Polar Lipids Inc., (Alabaster, AL, USA). Molecular sieves 4 Å and nylon filter (0.2 μm) were purchased from Sigma-Aldrich Corp. (St. Louis, MO, USA). Silicon Nitride cantilevers with spring constants 0.3 N/m were purchased from Bruker Inc. (Camarillo, CA, USA). The instruments used include a Branson 1510 sonicator bath, a white light interferometer (WYKO NT1100, Veeco, Tucson, AZ, USA, a surface profiler (TENCOR P10, KLA-Tencor, Milpitas, CA, USA), a Plasma-Therm 790 Series (Saint Petersburg, FL, USA), a scanning electron microscope (LEO1550, Pleasanton, CA, USA), an atomic force microscope (Pico Plus AFM 1550 from Molecular Imaging, Keysight Technologies, Santa Rosa, CA, USA), and an electrochemical impedance spectroscope (VersaSTAT MC by Princeton Applied Research, AMETEK, Berwyn, PA, USA.

### 2.2. Fabrication and Functionalization of Micron-Sized Cavity

A silicon-on-insulator (SOI) wafer was used with dimension 500:2:2.5 μm. A Si_3_N_4_ layer with a thickness of 2 μm was deposited on both sides using plasma enhanced chemical vapor deposition. This was followed by pattering a 1 mm × 1 mm square with SPR220 photoresist. Reactive ion etching was then performed to etch the exposed area of Si_3_N_4_. Next, a micron-sized silicon cavity of 20 μm × 20 μm was fabricated by immersing the chip in 30% KOH at 55 °C. A thin silicon membrane support with thickness of 2 μm was finally prepared by removing the SiO_2_ layer using 2% HF. The Si cavity chip was functionalized using 1% Triton X-100 (TX-100) [27] solution dissolved in phosphate buffered saline and incubated for 10 min. Detergent molecules were used to solubilize nanopore chip onto the surface through hydrophobic interactions and created half-micelle structures with the hydrophilic head group facing outward into the aqueous environment. The nanopore chip was washed with 0.1% TX-100 solution dissolved in PBS and dried overnight under germ free condition for air drying at room temperature. 

### 2.3. Preparation of Large Unilamellar Vesicles and Lipid Bilayer Membrane

Ampoules with lipids were dispensed in chloroform solution and the contents were transferred into pre-rinsed glass vials (2 mL). The vials were covered with teflon side screw cap and were stored at −20 °C. The final concentration of lipidic solution used in this work was 300 μL for DPPE (5 mg/mL) and 150 μL for DPPS (5 mg/mL). The solvent was evaporated under nitrogen for 15 min. The lipids were further dried in desiccator for 1 h under vacuum and rehydrated by adding the buffer comprising of 10 mM Tris and 10 mM NaCl. The large unilamellar vesicles (LUVs) were produced by sonication using a sonicator bath at room temperature for 40 min. Vesicles were then incubated for 20 min. The incubation temperatures were one slightly above (70 °C) and one below (50 °C) the *Tm* of DPPE and DPPS. Finally, the samples were cooled down to room temperature at the rate of 1 °C/min for the complete fusion and formation of SLBs. The sample was then rinsed with the buffer solution at room temperature several times in order to remove unfused vesicles attached to the membrane surface. 

### 2.4. Atomic Force Microscopy

The membrane imaging was performed in warm buffer comprised of 10 mM Tris and 10 mM NaCl using AFM nanoscope equipped with a J scanner (maximum XY scan range of 60 μm × 60 μm). The silicon cavity coated with lipid bilayer membrane was mounted in a fluid cell and was characterized in non-contact mode. A low force cantilever with a spring constant of 0.3 N/m was used to prevent any damage during scanning of the membrane surface. The cantilever oscillation was turned to a frequency of 120–180 kHz and the amplitude kept below a maximum of 6 nm. The scan rate was set to 5–10 Hz for imaging areas larger than 1 μm × 1 μm and was set to 10–22 Hz for smaller areas. The force applied on the sample was maintained at the lowest possible value by continuously adjusting the set point and gain during the imaging. Images were collected at a minimum of 256 × 256 pixels frames at different time intervals. The z-set point and differential gains were manually optimized during each scan. Images were analyzed and post-processed in the Gwyddion 2.31 software (http://gwyddion.net/).

### 2.5. Electrochemical Impedance Spectroscopy

Electrochemical impedance spectroscopy (EIS) was used to characterize the electrical properties in terms of resistance and capacitance as well as the stability of lipid bilayer membrane. In this work, the EIS measurements were recorded at frequency regime of 10^−2^ to 10^5^ Hz with signal amplitude of 100 mV. As shown in Figure S2, the Si cavity chip with pre-coated lipid bilayer was sandwiched between the two teflon chambers. The top chamber was filled with an electrolyte. O-rings were placed on each side of the chip to provide low stress on the cavity walls and to prevent any electrolyte leakage. Electrolytic solution (10 mM NaCl) was filtered using 0.2 μm nylon filter before adding to the top chamber. A platinum wire immersed in the electrolyte was used as the working electrode. The counter electrode along with reference was connected to Si chip via conductive tape. Measurements were recorded at different time intervals.

## 3. Results and Discussion

### 3.1. Fabrication of Micron-Sized Cavity in Si and Lipid Bilayers

**Figure 1:**
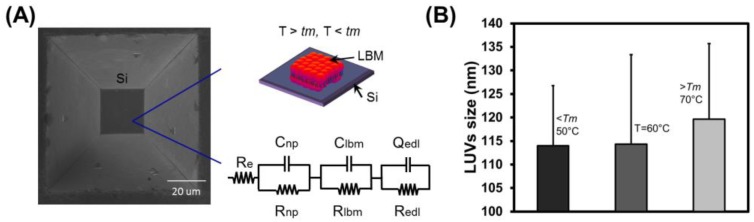
Anisotropically etched silicon with lipid bilayer membrane (LBM) produced using LUVs incubated at different temperatures. (**A**) The left shows a characteristic SEM image of a fabricated micron-cavity in Si. The right shows schematics of how the LBM is to be suspended in the cavity (top) and its equivalent electrical model (bottom); (**B**) Dynamic light scattering (DLS) measurements of LUVs incubated at different temperatures (50, 60, and 70 °C). Polydispersity index values obtained are below 0.3. Readings were obtained in triplicate and the error bars represent the standard error of the mean.

The thin base layer structures fabricated inside a silicon microstructure had cavity depths of ~493 ± 6 μm was fabricated using 30% KOH at 55 °C for four days followed by room temperature treatment for 4 h. A detailed description for the production of this silicon-based micron cavity was previously discussed in [28]. Figure 1 shows an image on the left from scanning electron microscopy (SEM) of a typical Si cavity fabricated in this work. The surface roughness inside the Si based cavity using AFM in tapping mode was 0.95 ± 0.11 nm. Low surface roughness plays an imperative role in the production of SLBs at below the *Tm* of the lipids. The schematic on the right represents how a bilayer film is to be suspended in the cavity (top) and how it is to be modeled for impedance spectroscopy (bottom).

Depending on several factors such as sonication time, incubation time, and temperature and cooling rate (Table S1), the sequence of events during SLB formation (adsorption, fusion, and spreading) were interpreted at different stages using AFM. When vesicles come into contact with a suitable solid surface, they may adsorb, break up, or spread to form lipid bilayer membrane on a hydrophilic surface or a monolayer on a hydrophobic one. Protrusion usually occurs in lipid bilayers formation from vesicles [3,29,30]. Sometimes high thickness can be seen due to the presence of trapped, adsorbed, and partially fused vesicles. Using AFM, the effect of protrusion can be captured and helpful to visualize the formation of SL at below the *Tm* of the lipids. Therefore, AFM imaging was performed by scanning the AFM probe across the surface of the preformed SLB, which provides information on the topographical characteristics of the supported lipid bilayer, such as the lateral extent of domains and height of patches relative to the substrate. We found that the time-scale of the interaction by imaging the surface after incremental time steps was revealed interesting information as described below. Size of the LUVs produced at different temperature was estimated using dynamic light scattering (DLS) as shown in Figure 1B. Here the size refers as hydrodynamic diameter of the individual vesicle. 

### 3.2. Real-Time Monitoring of SLB above Tm 

**Figure 2:**
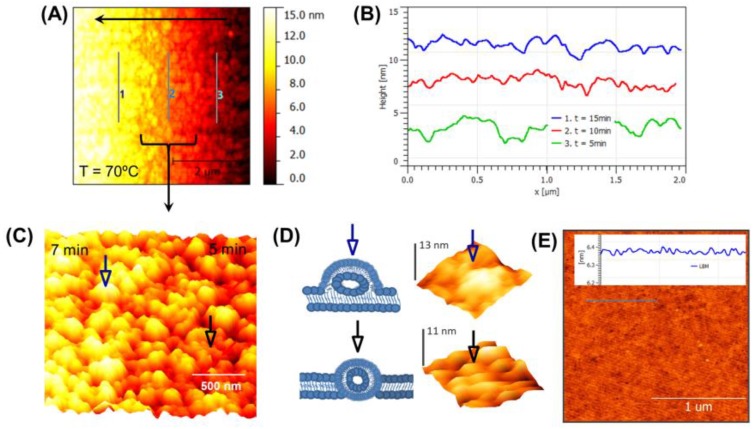
An illustration of real-time monitoring of a lipid bilayer membrane inside a Si-based cavity at 70 °C (above *Tm*) using AFM. (**A**) A single line profile at a fixed position across the membrane captured as a function of increasing time from right to left; (**B**) Individual line profiles extracted from the image on the left at the positions 3–1 corresponding to 5, 10, and 15 min; (**C**) High resolution shows that small patches formed instantaneously and fused to create the membrane. Blue arrow (partially fused flattened vesicle) and black arrow (trapped flattened vesicle) indicate distinguish characterization of vesicles at different states; (**D**) The left shows the schematic of perturbation due to partially fused flattened (top) and trapped (bottom) vesicles occurred during the formation of LBM. The right represents the 3D topography images of individual partially fused flattened and trapped vesicles with heights of 13 nm and 11 nm respectively; (**E**) An image of the LBM after it has been rinsed with warm buffer. A representative line profile across part of the image shows that characteristic hills or valleys (as observed in C) absent in the final SLB produced at above *Tm.*

Figure 2A shows in situ AFM images obtained in an aqueous solution for SLBs with coverage of 2 μm × 2 μm. In this case, LUVs were incubated at above (70 °C) the *Tm* of lipids (54 °C for DPPS and 65 °C for DPPE). Figure 2A reveals continuous flow at three stages for LUVs adsorption, fusion, and finally the production of lipid bilayer with partially fused and unfused flattened vesicles. Horizontal black arrow in Figure 2A indicates the scan direction of AFM tip from right to left and also represent the time information from 5 to 15 min with cooling rate (1 °C/min). Figure 2B illustrates the thickness profiles at different stages. At *t* = 5 min (line 3), the vesicles spontaneously fused to form irregular small patches of bilayer(s). At *t* = 10 min (line 2), lipid bilayer shows substantial unabsorbed and trapped vesicles with flattened shape. At *t* = 15 min (line 1), the membrane shows the complete coverage with presence of both partially fused and unfused flattened vesicles on top of the base membrane layer. Figure 2C (*t* = 5–7 min) illustrates the fusion procedure at high resolution with same height scale as in Figure 2A of lipid molecules. This image is an overview of the scan area showing three different stages, including attached liposomes, partly flattened vesicles, and membrane sheets. At *t* = 2 min, vesicles started accumulating and interacting with Si support to produce the base layer with some flattened trapped vesicles (Figure 2D bottom). At *t* = 7 min, both unfused and partially fused flattened vesicles were dominant over the original base layer of membrane. This represents that the partially fused vesicle was close to flattened shape (blue arrow) instead of spherical as illustrated in Figure 2D (bottom). This shows that a partly flattened liposome slowly collapses from the outer edges and forms a bilayer. The cross sections clearly show the decrease in height of the original vesicle from nearly 119 nm (Figure 1B) to the height of a double bilayer, 13 nm, as observed at the edges as shown in Figure 2D. The vesicles fused and spread immediately on contact with the silicon support followed by vesicle adsorption on top of the originally established SLBs. The unfused vesicles could be removed by rinsing with PBS (pH 7.4). Warm imaging PBS buffer was injected using syringe pump with flow rate of 50 μL/min. This successfully removed the remaining unbounded vesicles from the original intact membrane as shown in Figure 2E. Lipid bilayer thickness was estimated to be 4.9 ± 0.05 nm. An additional thickness revealed the surface roughness (0.94 ± 0.11 nm) of thin silicon membrane and the presence of thin hydrophilic layer (~1–2 nm) between the substrate and the membrane.

### 3.3. Real-Time Monitoring of SLB below Tm

**Figure 3:**
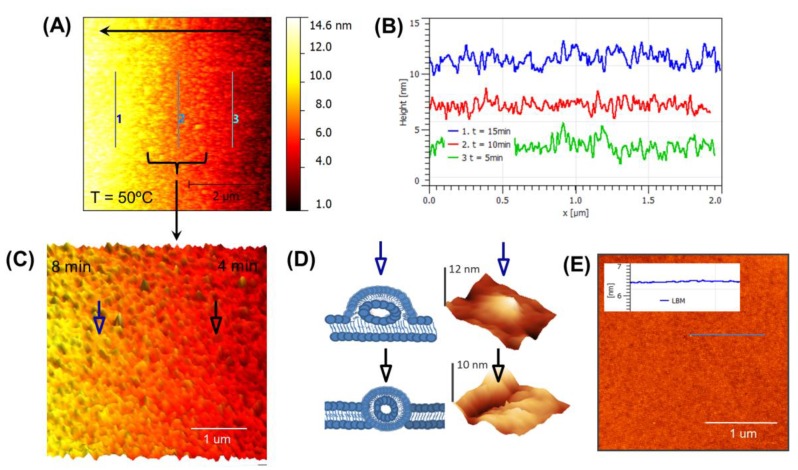
An illustration of real-time monitoring of a lipid bilayer membrane inside a Si-based cavity at 50 °C (below *Tm*) using AFM. (**A**) AFM imaging reveals the fusion of vesicles procedure from left to right across the formation of lipid bilayer; (**B**) The time accumulation of profile scans with AFM at the positions 3–1 corresponding to 5, 10, and 15 min; (**C**) High resolution shows that small patches formed instantaneously and fused to create the membrane. Blue arrow (partially fused flattened vesicle) and black arrow (trapped flattened vesicle) show distinguish characterization of vesicles at different stages; (**D**) Illustration of perturbation due to partially fused flattened (top) and trapped (bottom) vesicles occurred during the formation of LBM and the 3D topography images (right) represent heights of 12 nm and 10 nm, respectively; (**E**) An image and extracted line profile of the membrane after it has been rinsed with warm buffer. A representative line profile across part of the image shows that characteristic hills or valleys (as observed in C) are absent in the final SLB produced at below *Tm.*

Another experiment was conducted to monitor the fusion procedure of incubated vesicles inside the silicon-based micron-sized cavity. In this case, the incubated temperature was below (50 °C) the *Tm* of lipids. The results presented here fill a gap in understanding the events involved in formation of a planar, supported lipid bilayer from liposomes incubated at below the *Tm*. Figure 3A illustrates the formation of membrane with surface coverage of 2 μm × 2 μm. It reveals the stages for LUVs adsorption, fusion, and finally production of lipid bilayer with partially fused flattened and trapped flattened vesicles. Vesicles were instantly fused with substrate and formed random patches of bilayer as shown in line-profile of Figure 3B at different stages. The results suggest that once the process is initiated, it is a smooth and continuous chain of events. Some of the partially fused vesicles (blue arrow) and trapped vesicles (black arrow) were still intact within bilayer as shown in Figure 3c. This scan sequence (right to left) shown in Figure 3C illustrates the collapsing of a liposome onto itself and the intermediate stages in the formation of a bilayer membrane. As shown in Figure 3D, the partially fused (top) and trapped (bottom) vesicles were flattened from the outside toward the center until it completely collapsed onto itself. Also, some spreading takes place during this flattening process. The right represents the schematic of perturbation during the membrane formation and the left shows the individual image of the flattened fused and trapped vesicles with height of 12 nm and 10 nm, respectively. The dimensions of various structures present in the images were measured by cross section analysis performed in the fast scan direction of the height images. Estimations of the liposome sizes were made from attached liposomes under the imaging conditions outlined in the methods section. The height measurements may also be influenced by the tip forces, although they were minimized by using tapping mode. Unattached flattened vesicles were removed using imaging warm buffer with slow rinsing and the base lipid membrane showed complete coverage, even the vesicles were incubated successfully below their *Tm* as shown in Figure 3E. We have also studied the formation of SLB at 60 °C inside the silicon cavity and the results are presented in Figure S1.

### 3.4. Electrochemical Impedance Spectroscopy

The electrochemical method is a well-known technique to characterize the lipid bilayer supported on a substrate. The impedance response for electrochemical systems reflects the electrical characterization of a system under study and is commonly represented as electrical equivalent circuit. As shown in Figure 1A, the circuit is comprised of a simple electrolyte resistance (R_e_) in series with the parallel RC elements (R_Si_ and C_Si_). R_Si_ and C_Si_ are the resistance and capacitance of the silicon cavity support, respectively. Additional parallel RC elements (R_lbm_ and C_lbm_) are introduced in series with parallel RC elements of Si cavity (R_Si_ and C_Si_). R_lbm_ and C_lbm_ are the resistance and capacitance of the supported lipid bilayer produced at below the *Tm* of the lipids. There is an additional distribution of ions which reflects the non-ideal behavior of a system and is commonly expressed as constant phase element (CPE) [31,32]. Therefore, in the same equivalent circuit, additional parallel RC elements (R_edl_ and Q_edl_) are introduced in series with parallel RC elements of membrane (R_lbm_ and C_lbm_). Here, R_edl_ and Q_edl_ are the charge transfer resistance and capacitance of electric double layer (EDL) formed at the interface between the membrane and the electrolyte. Here, Q_edl_ is treated as Y_CPE_(ω) = Y_0_(iω)^α^ [31,32], where Y_0_ and *n* (0 < α < 1) are the CPE coefficient and exponent, respectively. Q_edl_ is estimated as a pure capacitance when is α close to 1 and a pure resistance when α is close to 0. 

**Figure 4:**
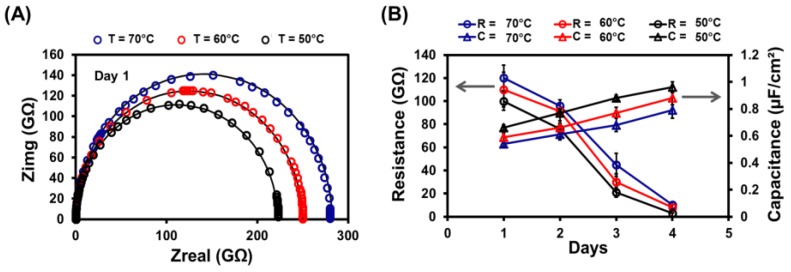
Measurements using EIS on an SLB in the micro-cavity at different temperatures (50, 60, and 70 °C). The Nyquist plot on the left reveals the impedance details of the pre-formed lipid bilayer. The solid black lines are the results of fitting the data (circles) using the electrical model presented in Figure 1A. The resistance (circles) and capacitance (triangles) values extracted from SLBs over different days for different temperatures.

Electrochemical characterization was done by assembling the Si cavity coated with lipid bilayer membrane in between two teflon chambers at the frequency regime of 10^−2^–10^5^ Hz with signal amplitude of 100 mV. Figure 4A shows the Nyquist plot of coated SLB inside Si cavity formed at different incubation temperatures (70, 60, and 50 °C). To extract the information for membrane impedance and capacitance, experimental data were characterized using equivalent circuit as shown in Figure 1A. The membrane capacitance of 0.71 ± 0.053, 0.65 ± 0.034, and 0.61 ± 0.011 μF/cm^2^ was obtained after fitting data for 50, 60, and 70 °C respectively, as shown in Figure 4B. This data is consistent with lipid membranes produced on ordered porous silicon membrane (0.7 ± 0.3 μF/cm^2^) [33], nonordered porous silicon membrane (0.63 μF/cm^2^) [34,35], ordered porous alumina (0.65 ± 0.2 μF/cm^2^) [36], and porous silicon nitride (0.40 μF/cm^2^) [37]. The capacitance obtained in this work is in agreement with those obtained for the closely related phospholipid phosphatidylcholine membrane in a 1 mM NaCl electrolyte and was reported to be ~0.62 μF/cm^2^ [38,39]. 

In this study, the membranes exhibited >1 GΩ sealed resistance with at least a four-day-long life time (*n* = 11, *n* represents number of trials). After performing the data fitting, the impedance of the SLB was estimated to be 121.11 ± 10.26, 108.22 ± 10.33, and 101.68 ± 9.18 GΩ for 70, 60, and 50 °C respectively as shown in Figure 4B. Previously, high impedance (26.3 GΩ) of SLB was reported by controlling the contact area between the aqueous droplet and the conductive solid surface in an oil solution containing phospholipids [40]. In this work, we present SLB produced inside Si cavity at below *Tm* exhibited almost four times more GΩ sealed impedance. We also estimated the resistance (R_edl_) and capacitance (Q_edl_) occurred due to the presence of electric double layer (EDL) formed at the interface of the membrane and the electrolyte. This results in R_edl_ of 1.6 KΩ and Q_edl_ of 0.796 nF-s^α−1^ (α = 0.781). Figure S3 represents the magnitude plot using EIS results for the SLB produce at 50 °C (below *Tm)* over period of time.

### 3.5. Stability of Lipid Bilayer Membrane

Long-term stability of SLB is one of the prerequisites for a robust biosensor and high throughput drug screening [40,41,42]. The initial impedance for SLB produced at below *Tm* was recorded to be 101.68 ± 9.18 GΩ. The membrane resistance was gradually dropped to 78.24 ± 9.06 and 22.38 ± 4.25 GΩ during intervals of 48 h and 72 h, respectively. After four days, membrane exhibited the reasonable resistance of ~1.25 GΩ (*n* = 21) as shown in Figure 4B. Comparably, the most successful research article for SLB produced using small unilamellar vesicles (SUVs) exhibited a life of membrane for about 50 h [41]. They utilized the expensive high energy electron beam tool to fabricate the pore in Al_2_O_3_ support and then produce SLB. Later, to reduce the cost of a substrate material and a fabrication method, the set of tapered apertures with different shapes (beak, triangle, and cylindrical) were fabricated [42]. They developed apertures in SU8 photoresist with the diameter ranging from 60 to 80 μm. Instead of creating supported lipid bilayer, they produced black lipid membranes (BLMs) by employing painting and folding techniques with the life of 36 h (triangle shaped aperture) and 21 h (beak-shaped aperture), respectively. Results obtained in this work utilized Si micron-sized cavity as a support to produce SLB with the stability of about 96 h as shown in Figure 4B. 

### 3.6. Force Spectroscopy Study of SLB below Tm

**Figure 5:**
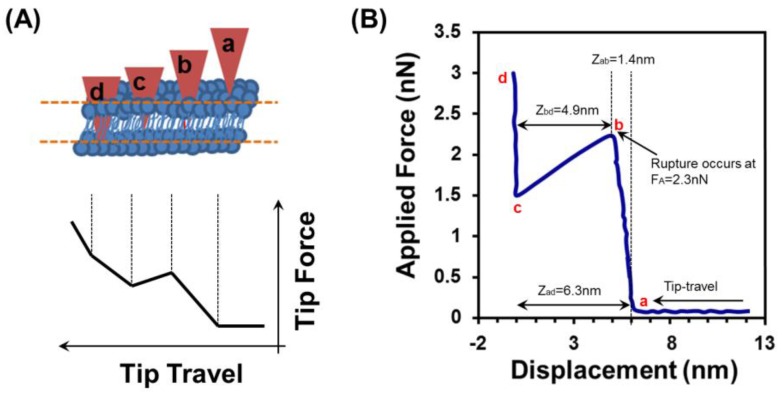
Force spectroscopy study of SLB produced at below *Tm*. (**A**) An illustration of the steps taken to measure the mechanical properties of a bilayer. The tip travels through the bilayer (top schematic) as an increasing force is applied to it. The force is measured as a function of travel through the layer (bottom schematic). The physical significance is interpreted as follows: (a) First contact of the tip with the top surface of the bilayer; (b) Rupture of the upper surface of the bilayer, (b–c) Rapid tip transition through the central portion of the bilayer; (c) On-set of increased repulsion associated with compression of proximal head groups, water layer and other trapped material; (c–d) Compression of trapped material; (d) Tip in direct contact with silicon surface inside the cavity; (**B**) A representative measurement of force–distance from a preformed SLB inside the Si cavity at below *Tm*. Rupture of the top layer occurs at a force of 2.3 nN after a tip displacement of 1.4 nm from first contact. The tip reaches the Si surface at 6.3 nm displacement.

To demonstrate force spectroscopy study for the SLB produced below the *Tm* inside the micron-sized Si cavity, the cantilevers’ tip approaches and leaves the surface in a repetitive manner. The force experienced by the cantilever can be detected and then plotted against the z-piezo displacement. During measurement, AFM probe was brought towards the SLB and a load increasingly applied until the bilayer ruptures. Afterwards, the probe was withdrawn; the cycle was repeated many times. In our work, the force experienced by the cantilever was detected and then plotted against the tip-sample separation (displacement). Figure 5 describes the effect of tip cantilever on the surface of the SLB produced at below *Tm* inside the silicon cavity. 

Figure 5A depicts the scheme of a cantilever deflection with respect to depth of the lipid bilayer membrane. Initially, there is no cantilever deflection until reaching point ‘a’ and then a sudden jump-to-contact occurs at just beyond point ‘a’, resulting in a tip position at point ‘b’. Increasing the pressure force to the membrane, AFM cantilever deflection reveals the linear response to piezo displacement. This provides a linear relationship between the applied force and the piezo, as shown in line ‘a–b’ of Figure 5A. Upon reaching point ‘b’, a sudden jump to point ‘c’ occurs, which represents the actual breakthrough of the lipid bilayer membrane. Further increasing the force leads to an apparent linear relationship (c–d), which contains valuable information since the tip pyramid has been in contact with the substrate. Here Point ‘a’ is not always well-defined, for example, due to long range electrostatic repulsion leading to a curved region around ‘a’, and a similar problem may be observed around ‘d’. 

**Figure 6:**
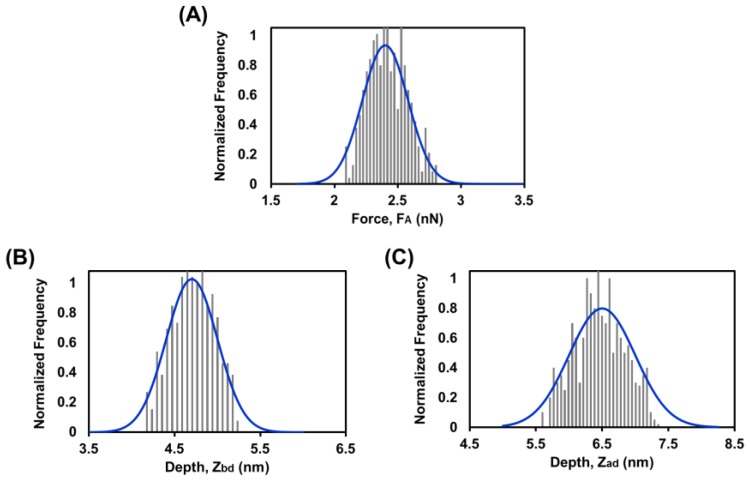
Supported lipid membrane rupture force and depth distributions. (**A**) Rupture force, F_A_; (**B**) Z_bd_; (**C**) Z_ad_. Mean values are F_A_ = 2.4 ± 0.18 nN, Z_ad_ = 6.5 ± 0.4 nm, and Z_bd_ = 4.7 ± 0.3 nm (errors are standard deviations). Blue lines are Gaussian fits to the distributions.

In the real-time force spectroscopy experiment, a single representative force–distance curve for the rupture of SLB was derived as shown in Figure 5B The z-piezo displacement was converted to tip-sample separation so as to more accurately reflect the tip dynamics. The first cantilever deflection occurred at ‘a’ and with an immediate jump, the tip moved to ‘b’. This most likely happens due to electrostatic attraction between the tip and the lipid bilayer membrane. As displayed in Figure 5B, the tip was first interacted with the upper surface of the bilayer at a distance Z_ad_ = 6.3 nm from the silicon surface and then rupture was occurred at F_A_ = 2.3 nN with depth Z_bd_ = 4.9 nm. In order to gain a more accurate reflection of the bilayer properties, the force–distance measurements were repeated several times. As shown in Figure 6, the data represents the approach–retract cycles for a single tip. The mean values are F_A_ = 2.4 ± 0.18 nN, Z_ad_ = 6.5 ± 0.4 nm, and Z_bd_ = 4.7 ± 0.3 nm. This Z_bd_ depth is in good agreement with the other reports in the literature [1,43,44]. 

## 4. Conclusions

The work presented in this paper reveals new study for the production of a supported lipid bilayers, SLBs on an anistropically etched micron-sized cavity in silicon at above and below the phase transition temperature, *Tm* of the lipids (DPPE and DPPS). Initially, a micron-sized cavity was fabricated using standard microfabrication procedure. Large unilamellar vesicles were prepared and incubated at above and below the *Tm* of lipids. By using continuous flow mode of atomic force microscopy in natural fluidic environment, all major steps (adsorption, fusion, and formation) for the formation of lipid bilayer including vesicles attachment were monitored in real-time environment, captured, and discussed. We demonstrated that SLBs can also be formed without attaining the deposition temperature above *Tm* of the lipids, but subsequent cooling effects on the final membranes’ structure need to be taken into account. Stability and electrical properties of the SLBs produced at different temperatures were characterized and recorded continuously over four days using electrochemical impedance spectroscopy with a three electrode system. Impedance and capacitance of the SLBs were compared with previously reported data and the results are in good agreement. Finally, the rupture force and the bilayer depth were estimated using force spectroscopy measurements. We anticipate that a silicon based micron-sized cavity could be helpful in preparing lipid bilayer membranes at below *Tm* for nano-medicine applications. 

## Supplementary Materials

The following are available online at www.mdpi.com/2079-6374/7/3/26/s1, Figure S1: An illustration of real-time monitoring of a lipid bilayer membrane inside a Si-based cavity at 60 °C using AFM, Figure S2: Schematic assembly of silicon-based micron-sized cavity with teflon chamber for EIS studies, Figure S3: The magnitude plots for supported lipid bilayers produced at 50 °C (below *Tm*) over period of time, Table S1: Summarized results for successful preparation of lipid bilayer membranes inside silicon-based micron-sized cavity at different incubation temperatures.
