# Supplementary material for: Lipid Bilayer Membrane in a Silicon Based Micron Sized Cavity Accessed by Atomic Force Microscopy and Electrochemical Impedance Spectroscopy
Source: Biosensors (Basel). 2017 Jul 5;7(3):26. doi: 10.3390/bios7030026 (PMC5618032; doi:10.3390/bios7030026)
Supplement: Supplementary file 1 [file biosensors-07-00026-s001.doc]

**Electronic Supplementary Information**

**Lipid Bilayer Membrane in a Silicon-Based Micron-Sized Cavity Accessed by Atomic Force Microscopy and Electrochemical Impedance Spectroscopy**

Muhammad Shuja Khan ^1^,*, Noura Sayed Dosoky ^2^, Darayas Patel ^3^, Jeffrey Weimer ^4^ and John Dalton Williams ^1^

^1^ Electrical and Computer Engineering Department, University of Alabama in Huntsville, Huntsville AL USA 35899

^2^ Biotechnology Science and Engineering Program, University of Alabama in Huntsville, Huntsville AL USA 35899

^3^ Department of Mathematics and Computer Science, Oakwood University, Huntsville AL USA 35896

^4^ Chemistry / Chemical and Materials Engineering Department, University of Alabama in Huntsville, Huntsville AL USA 35899

***** Correspondence: [mskhan7@illinois.edu](mailto:mskhan7@illinois.edu); Tel.: +1-256-929-5572

**Abstract:** Supported lipid bilayers (SLBs) are widely used in biophysical research to probe the functionality of biological membranes and to provide diagnoses in high throughput drug screening. Formation of SLBs at below phase transition temperature (*Tm*) has applications in nano-medicine research where low temperature profiles are required. Herein, we report the successful production of SLBs at above as well as below the *Tm* of the lipids in an anisotropically etched, silicon-based micro-cavity. The Si-based cavity walls exhibit controlled temperature which assist in the quick and stable formation of lipid bilayer membranes. Fusion of large unilamellar vesicles was monitored in real time in an aqueous environment inside the Si cavity using atomic force microscopy (AFM), and the lateral organization of the lipid molecules was characterized until the formation of the SLBs. The stability of SLBs produced was also characterized by recording the electrical resistance and the capacitance using electrochemical impedance spectroscopy (EIS). Analysis was done in the frequency regime of 10^-2^ – 10^5^ Hz at a signal voltage of 100 mV and giga-ohm sealed impedance was obtained continuously over four days. Finally, the cantilever tip in AFM was utilized to estimate the bilayer thickness and to calculate the rupture force at the interface of the tip and the SLB. We anticipate that a silicon-based, micron-sized cavity has the potential to produce highly-stable SLBs below their *Tm*. The membranes inside the Si cavity could last for several days and allow robust characterization using AFM or EIS. This could be an excellent platform for nanomedicine experiments that require low operating temperature.

**Table of Contents:**

**Figure S1:** An illustration of real-time monitoring of a lipid bilayer membrane inside a Si-based cavity at 60°C using AFM.

**Figure S2:** Schematic assembly of silicon-based micron-sized cavity with Teflon chamber for EIS studies.

**Figure S3:** The magnitude plots for supported lipid bilayers produced at 50°C (below *Tm*) over period of time.

**Table S1:** Summarized results for successful preparation of lipid bilayer membranes inside silicon-based micron-sized cavity at different incubation temperatures.

**
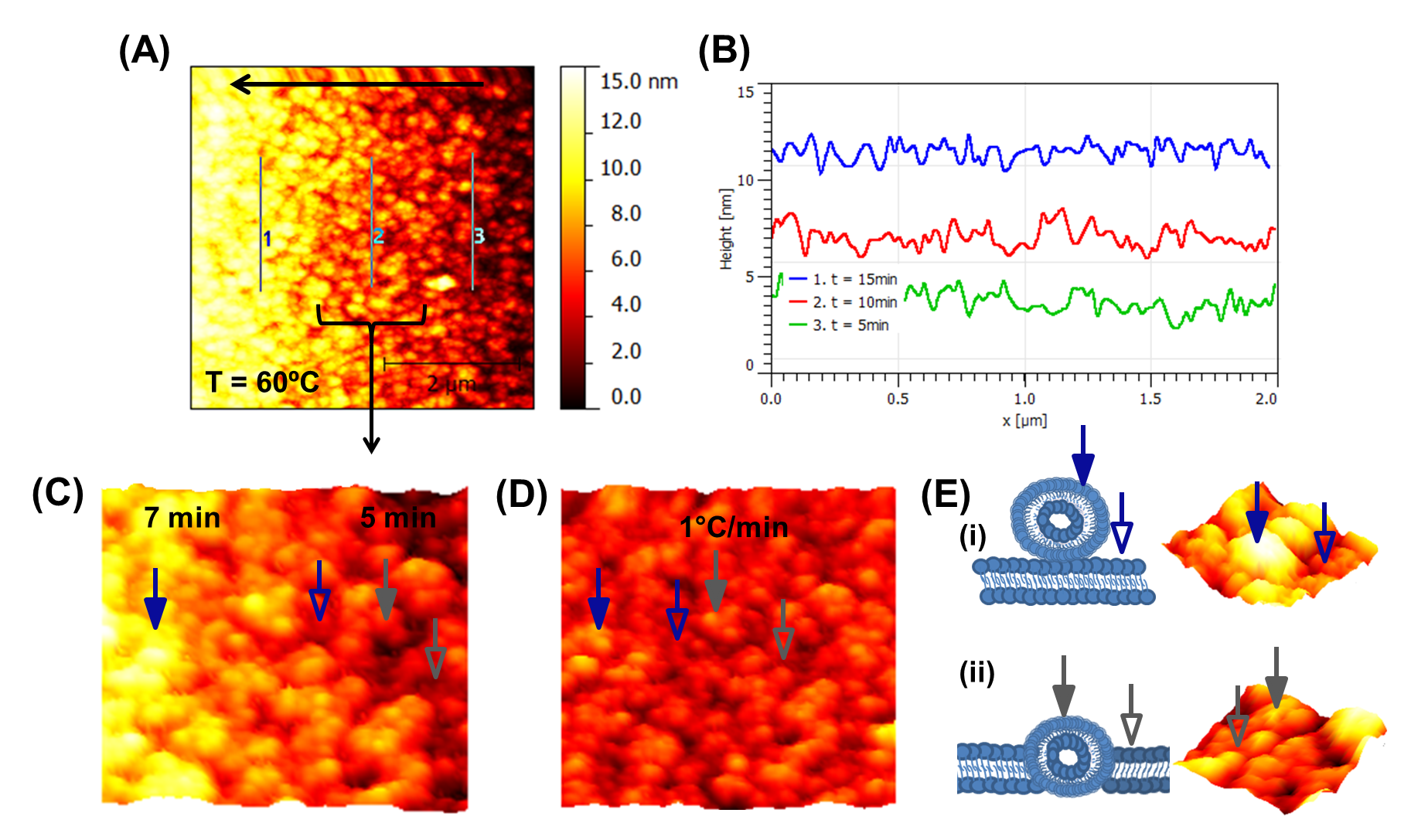
**

**Figure S1.** An illustration of real-time monitoring of a lipid bilayer membrane inside a Si-based micron-sized cavity at 60°C using AFM. (A) AFM imaging reveals the fusion of vesicles procedure from left to right across the formation of lipid bilayer. (B) The time accumulation of profile scans with AFM at the positions 1 - 3 corresponding to 5 min, 10 min, and 15 min. (C) High resolution AFM data shows the formation of small patches distributed close to each other. Blue (adsorbed) and gray (trapped/partially fused) arrows indicate different features of vesicles at different states. (D) SLB at RT with rate 1°C/min. (E) Illustrations and 3-D expansions at the marked positions in the left image to show the base membrane (open arrows) and either a adsorbed vesicle (top - solid arrow) or trapped vesicle (bottom - solid arrow).

**
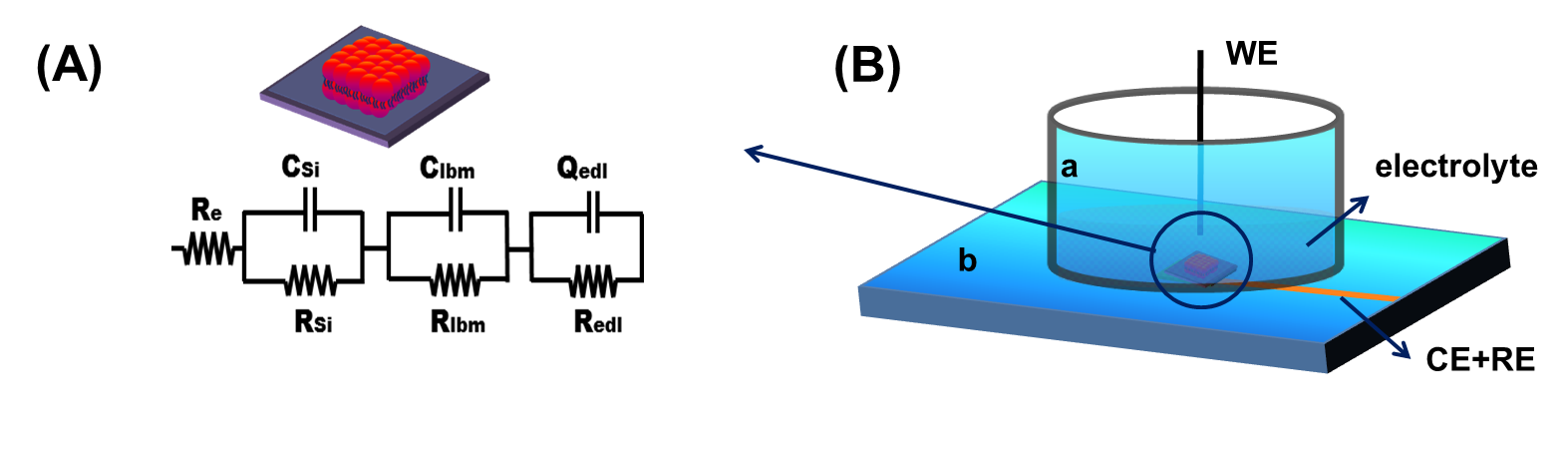
**

**Figure S2.** Electrochemical impedance spectroscopy study of lipid bilayer membrane formed inside the silicon cavity at above and below the phase transition temperature of lipids (DPPE, DPPS). (A) Electrical equivalent model used for data fitting to extract the information of resistance and capacitance for LBM. (B) Schematic represents the experimental setup utilized to perform EIS. Pre-coated silicon chip was assembled carefully in between two Teflon based supports (‘a’ and ‘b’).


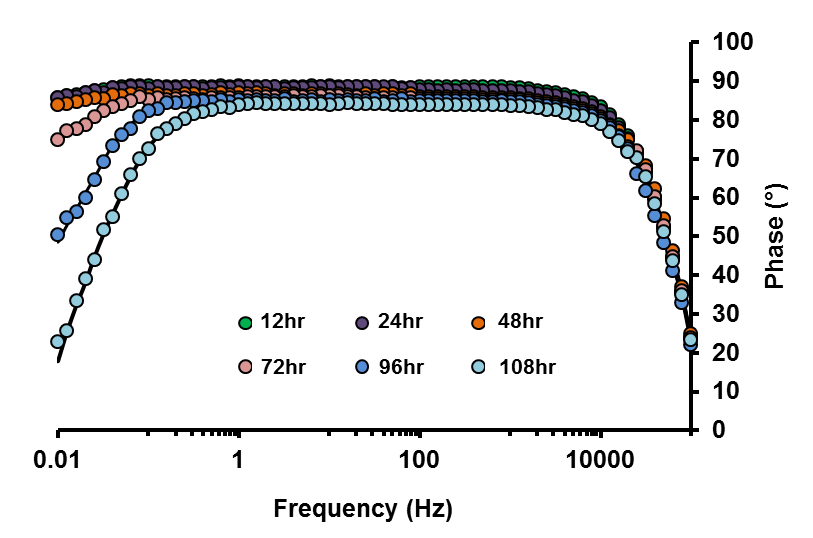


**Figure S3.** The magnitude plots for supported lipid bilayers produced at 50°C (below *Tm*) over period of time.

**Table S1:** Summarized results for successful preparation of lipid bilayer membranes inside silicon-based micron-sized cavity at different incubation temperatures.

| **LUVs Sonication Time (min)** | **Incubation Temperature (ºC)** | **Incubation Time (min)** | **Cooling Rate (ºC/min)** | **Remarks** |
| --- | --- | --- | --- | --- |
| 40 | 80 | 15 | 1.25 | No success |
| 40 | 70 | 20 | 1.0 | Success |
| 40 | 60 | 20 | 1.0 | Success |
| 40 | 50 | 20 | 1.0 | Success |
| 40 | 45 | 25 | 0.75 | partial success |
| 50 | 40 | 30 | 1.0 | No success |
